# Three-Dimensional Assessment of Morphological Changes Following Nasoalveolar Molding Therapy in Cleft Lip and Palate Patients: A Case Report

**DOI:** 10.3390/dj7010027

**Published:** 2019-03-07

**Authors:** Edoardo Staderini, Romeo Patini, Andrea Camodeca, Federica Guglielmi, Patrizia Gallenzi

**Affiliations:** Institute of Dentistry and Maxillofacial Surgery, Università Cattolica del Sacro Cuore, Fondazione Policlinico Universitario A. Gemelli IRCCS, Largo A. Gemelli, 8, 00168 Roma, Italy; edoardo.staderini@yahoo.it (E.S.); andrecamo@tiscali.it (A.C.); fe.guglielmi@gmail.com (F.G.); patrizia.gallenzi@unicatt.it (P.G.)

**Keywords:** cleft lip and palate, stereophotogrammetry, case report

## Abstract

The applications of computer-guided technologies for three-dimensional image analysis provide a unique opportunity to quantify the morphological dimensional changes of the face in a practical and convenient way. Symmetry of the nasolabial area is one of the main factors of facial attractiveness as well as being the main objective of the treatment of cleft lip and palate (CLP). Technological advances in computer-guided visualization modes and their applications to three-dimensional stereophotogrammetry provide more practical opportunities and alternatives for facial analysis. Each study, however, uses different protocols for the acquisition and analysis of three-dimensional images. In addition, each study identifies different anthropometric points and calculates linear and angular measurements with overlapping protocols. Therefore, it is appropriate to define a standardization of the three-dimensional analysis of CLP patients to compare the studies of different research centers. The aim of this report is to propose a protocol to standardize the acquisition and analysis of three-dimensional images to evaluate the three-dimensional changes in the nasolabial area in cleft lip and palate patients undergoing pre-surgical nasoalveolar molding (PNAM).

## 1. Introduction

Pre-surgical nasoalveolar molding (PNAM) treatment before primary lip repair was introduced by McNeil in 1956 to provide stimulation and guidance to maxillary segment growth as well as to facilitate surgical management of cleft lip and palate (CLP) patients [1].

It has been reported that NAM treatment can mold the deformed nasal cartilage into a more symmetrical form and position and lengthen the deficient columella [2,3].

There are several techniques to analyze nasolabial soft tissue changes following NAM therapy: direct anthropometric measurements, photometric assessment, cephalograms, and cone beam computed tomography (CBCT) scans [4,5,6].

It is intuitive and demonstrated in literature that stereophotogrammetry is a noninvasive gold-standard technique for the evaluation of the qualitative and quantitative effects on soft tissues of the orofacial region [7]. Stereophotogrammetry offers a better reproducibility and higher efficacy than standard two-dimensional (2D) cephalograms or photographs [8].

Many studies document three-dimensional (3D) facial changes induced by PNAM, however there is an absence of systematic reviews specifically investigating the topic. Therefore, it is necessary to introduce a standardized protocol to quantify soft-tissue nasolabial labial changes and to compare the different treatment options. [9].

## 2. Case Report

This study refers to a patient with a complete unilateral cleft lip and palate (Veau class III) on the left side, not associated with syndromic spectrum.

The patient was not subjected to any type of intervention before being included in the study.

At the time of first access, anamnestic data were collected, an objective examination was performed, and pre-surgical orthopedic therapeutic treatment was planned. Parents were informed that pre-surgical orthopedic treatment should be performed, explaining the risks and potential benefits of therapy. Upon being informed about the use and the hygienic maintenance of the oral device, the parents signed the consent form, without which the therapeutic plan could not be executed [10,11].

The first plate was delivered 16 days after birth. The last plate was used until 6 months of age, when the patient underwent lip surgery.

The active plates were used to reduce the transverse discrepancy between the emimaxillary segments before surgery.

When the width of the alveolus cleft is reduced to about 5 mm, the clinician decided to realize a passive plate to avoid unfavorable repositioning of the maxillary bone segments [2,3].

The treatment lasted 8 months and the mean duration of use of each plate was about 2 months. A total of four devices were used:

- Two active plates, in combination with adhesive Figueroa lip taping [12], aimed at achieving a transverse dimension of the cleft associated with nostril modeling;
- One active plate, with nasal stent and lip taping, to achieve columella repositioning and the nasal modeling simultaneously with alignment of the alveolar processes;
- One passive plate, with nasal stent and lip taping, to optimize nasal symmetry and stabilize the clinical results obtained.

**Figure 1:**
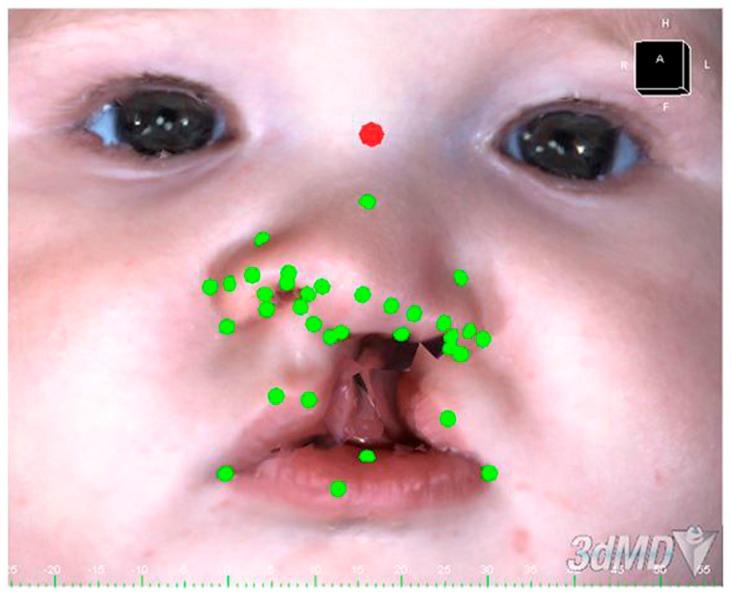
Three-dimensional acquisition of the face with anthropometric points.

The three-dimensional photographs were obtained using the 3dMD system (3dMD, Inc, Atlanta, GA, USA) before the delivery of the orthopedic equipment and 1 month after PNAM treatment. The authors evaluated qualitative and quantitative changes in nasoalveolar asymmetry using Geomagic Studio^®^ 2014 software (Figure 1).

### Two- and Three-Dimensional Analysis

The study refers to anthropometric points, with linear, curvilinear, and angular measurements as described by Singh et al. [13].

To guarantee the reproducibility of the results, all analyses were performed by the same expert operator.

The study analyzed the reduction of the asymmetry index, as described by Wu et al., because of the simplicity and high reproducibility of the protocol proposed by the authors [14].

**Figure 2:**
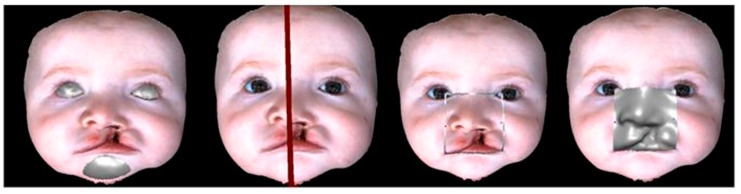
The digital workflow in a sequence shows: a selection of the symmetry areas; creation of the symmetry plan; delimitation of the area to be analyzed; and the overlap of a mirrored hemiface.

Through the selection of eyes and chin (Figure 2), the Geomagic software automatically calculates the ideal symmetry plane [15].

Then, the nasolabial area between the tangent line to the chelion and the tangent to the endocanthion is selected and “mirrored” along the symmetry plane; the distance between each point of the mirrored side and the nearest point in the original side is then calculated. The average between these distances (approximately 3000 points) represents the asymmetry index:

$$PD=\frac{1}{n}\sum_{p\;in\;the\;area}distance(p_{s},q)$$

The data were analyzed using a *t*-test in SPSS Statistics (version 20, IBM, Armonk, NY, USA).

The asymmetry index revealed a noticeable improvement of the facial symmetry after NAM therapy (Table 1).

The analysis by 3D overlap revealed a significant improvement in the symmetry of the nasolabial area obtained with the pre-surgical orthopedic treatment.

**Figure 3:**
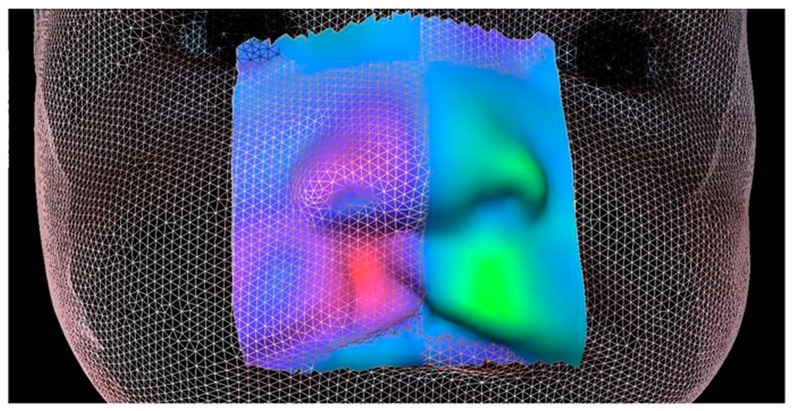
Overlap 3D of the pre- and post-treatment faces.

In the colormap display, warm colors are associated with an advancement area, while cold colors are associated with a retrusion (Figure 3).

The linear measurements (Table 1) disclosed that the affected nostril appeared to have shrunk in width (Sball-Sn’l), remaining larger than higher (Sbalr-Sn’r). The transverse width of the base of the nose decreased during treatment (Alr-All).

As far as angular measurements are concerned, an increase in the projection of the nose from 4.5° (Pm-Stn-Sn) to 20° (Sn-Prn-Stn) is shown. There is a noticeable slight decrease in the concavity of the nose wing on the cleft side, as indicated by the Sn-Prn-Adl value.

Likewise, angular measurements related to the transverse width of the nostrils and to the projection of the nose show a notable reduction both on the healthy side (Sn-Prn-Sbalr) and on the affected side (Sn-Prn-Sbalr).

## 3. Discussion

The application of computer-guided technologies for 3D image analysis provides a unique opportunity to quantify the morphological-dimensional changes of a face in a practical and convenient way. Unfortunately, only one example is given, so further research is needed to generalize our results.

The main advantage of the three-dimensional technology is that it provides a visual feedback of the treatment progress. It is difficult with the photometric and the human perception to rate a symmetry gain with the same level of accuracy.

Secondly, the report enhances the immediateness of the asymmetry index, which improves the efficiency of the clinicians understanding. Rather than focusing on single facial features, a single value makes the evaluation of the symmetry improvement and the comparisons between different treatment protocols intuitive.

Thirdly, caregivers must cope with the stress linked to the growth of a child with a craniofacial malformation, often linked to feelings of guilt, anxiety, anger, and depression [16,17].

Moreover, pre-surgical orthopedic therapy emphasized the patient-experienced burden of treatment; many parents begin pre-surgical orthopedic therapy thinking that they will do their best, but if the treatment is too dangerous and will cause too much stress, they may suspend the treatment [18]. With the widespread use of technology in everyday life, the 3D facial imaging is a gold standard to capture the audience’s attention. In the future it is possible that the display of 3D facial imaging will provide the rater with cues for depth perception of the facial symmetry gain and therefore incentivize parents towards better compliance.

## 4. Conclusions

The authors proposed a protocol to standardize the acquisition and analysis of 3D images to evaluate the three-dimensional changes in the nasolabial area in cleft lip and palate patients undergoing pre-surgical orthopedic plates.

Overall, the use of new 3D technologies is providing a positive contribution to the study of growth and treatment effects in newborns with malformation problems.

## Figures and Tables

**Table 1:** Two-dimensional face measures and asymmetry index.

Distances	Linear Measurements (mm)			Angles	Angular Measurements (Degrees)		
Values	Before NAM therapy		After NAM therapy	Values	Before NAM therapy		After NAM therapy
Stn-Sn	24.73	>	24.47	Acr-Sn-Prn	131.7	<	152.49
Stn-Prn	20.49	<	21.45	Acl-Sn-Prn	54.67	<	62.69
Sn-Prn	6.2	<	6.95	Sn-Prn-Acl	110.45	>	97.03
Sbalr-Sn’r	4.56	>	4.24	Sn-Prn-Acr	29.9	>	16.59
Sball-Sn’l	18.41	>	15.68	Sn-Prn-Alr	45.92	>	34.31
Alr-All	25.61	<	28.77	Sn-Prn-All	114.19	>	104.89
Acr-Acl	29.85	>	27.91	Sn-Prn-Adr	57.49	>	50.44
Sbalr-Sball	24.38	>	22.63	Sn-Prn-Adl	109.9	>	108.14
Sn-C’r	5.53	<	5.86	Sn-Prn-Sbalr	33.23	>	21.1
Sn-C’l	9.19	<	10.67	Sn-Prn-Sball	104.08	>	96.8
Acr-Prn	14.66	<	16.95	Prn-Stn-Sn	11.52	<	15.7
Acl-Prn	19.7	>	17.81	Sn-Prn-Stn	127.18	>	107.64
Asymmetry index		Mean		Standard deviation		*t*-Test	
Before NAM therapy		−3.167 mm		2.28 mm		<0.05 *	
After NAM therapy		1.57 mm		2.189 mm		<0.05 *	

**NAM** = nasoalveolar molding; **Sn** = subnasal point; **Sn’l** = intersection of the left side of the columella with the labial filter; **Sn’r** = intersection of the right side of the columella with the labial filter; **Sbalr** = intersection of the right side of the columella with the labial filter; **Sball** = left subarray point; **Alr** = right wing point; **All** = left wing point; **Acr** = point of the right wing bending; **C’r** = point of the right columella; **C’l** = point of the left columella; **Acl** = point of the left wing bending; **Adr** = right wing point; **Adl** = left wing point; **Stn** = soft tissue nasion; **Prn** = prostasal point. > changes related to an improvement of symmetry. < changes related to a worsening of symmetry. * = Statistically significant values.
